# Unraveling the Formation Mechanism of Egg’s Unique Flavor via Flavoromics and Lipidomics

**DOI:** 10.3390/foods13020226

**Published:** 2024-01-10

**Authors:** Zheng Zhou, Shuang Cui, Jing Che, Yuying Zhang, Dayong Zhou, Xuhui Huang, Lei Qin

**Affiliations:** State Key Laboratory of Marine Food Processing & Safety Control, School of Food Science and Technology, National Engineering Research Center of Seafood, Dalian Polytechnic University, Dalian 116034, China; zhouzheng971201@163.com (Z.Z.); cuishuang0327@163.com (S.C.); chejing99@163.com (J.C.); zhangyuying99210@163.com (Y.Z.); zdyzf1@163.com (D.Z.); xuhuisos@dlpu.edu.cn (X.H.)

**Keywords:** key flavor compounds, volatile compounds, lipidomics, flavor precursor

## Abstract

Egg products after thermal treatment possess a unique flavor and are favored by consumers. In this study, the key aroma-active compounds of egg yolk products and their formation mechanism during thermal treatment were investigated. The volatile aroma compounds in egg yolks were monitored using an electronic nose, gas chromatography-mass spectrometry (GC–MS) and gas chromatography–olfactometry–mass spectrometry (GC–O–MS), and the lipid molecular species were explored using ultra-high-performance liquid chromatography– mass spectrometry with a Q-Exactive HF-X Orbitrap (UPLC-Q-Exactive HF-X). A total of 68 volatile compounds were identified. Boiled eggs mainly derived their flavor from hexanal, 2-pentyl-furan, 2-butanone, 3-methyl-butanal and heptane. Meanwhile, fried eggs relied mainly on 14 compounds, the most important of which were 2-ethyl-3-methyl-pyrazine, 3-ethyl-2,5-dimethyl-pyrazine, 2-ethyl-3,5-dimethyl-pyrazine, nonanal and 2,3-diethyl-5-methyl-pyrazine, providing a baked and burnt sugar flavor. A total of 201 lipid molecules, belonging to 21 lipid subclasses, were identified in egg yolks, and 13 oxidized lipids were characterized using a molecular network. Phosphoethanolamines (PEs) containing polyunsaturated fatty acids were the primary flavor precursors contributing to the development of egg yolks’ flavor, participating in lipid oxidation reactions and the Maillard reaction and regulating the production of aldehydes and pyrazine compounds. This study provides reference and guidance for the development of egg yolk flavor products.

## 1. Introduction

Eggs are one of the most popular animal-origin foods due to their low price, rich nutrition and delicious flavor. Most people eat them regularly, as they are rich in phospholipids (PL), essential fatty acids, amino acids, minerals, vitamins and high-quality protein [1]. Egg yolks are a rich source of phospholipids, accounting for 22% of their total lipids [2,3]. Moreover, eggs can be processed using a variety of cooking techniques, yielding a wide array of egg-based dishes such as egg tarts, fried eggs and boiled eggs, which all possess good flavors. The unique egg aroma can stimulate consumers’ sense of smell to increase the attractiveness of egg products. Although some researchers have studied the flavor of eggs in recent years, most of them have focused on raw eggs, with a few analyzing salted eggs [4,5]. The egg aroma produced using thermal treatment has been widely recognized, but the related studies were carried out 20 years ago, with longer heating and extraction times due to technological limitations at that time [6,7,8]. For example, Umano et al. used a simultaneous purging–extraction apparatus to collect the volatile flavor compounds formed in heated egg samples, which required the egg yolks to be heated at 200 °C for one hour [7]. This may result in differences between the flavor in previous studies and the actual flavor of heat-processed eggs in real life. Moreover, previous studies have also lacked coordinated sensory research, such as gas chromatography–olfactometry, which did not provide a clear identification of the key flavor volatile compounds [6,7,8]. The absence of research on the formation mechanism of egg’s flavor also limits the ability to control egg’s flavor and its application in food production.

Egg yolk is recognized as the primary source of egg’s flavor owing to its high-fat content, which accounts for almost all of the total fat present in eggs [9]. Notably, lipid oxidation is a critical determinant of food quality attributes, especially for animal-based products like eggs. It exerts a profound impact on food’s flavor. Typically, lipid oxidation and degradation are responsible for more than half of the volatile compounds in animal foods. The aldehydes, alcohols, acids, ketones, esters, etc. produced by lipid oxidation are often thought to cause the meat aroma characteristic of animal foods [10]. Unsaturated aldehydes with a low odor threshold, such as 2,4-decadienal, provide a strong fried flavor at relatively low concentrations [11]. Many researchers are interested in exploring the relationship between lipids and flavor compounds [12,13,14]. Zhou et al. investigated the effects of different fatty acid chains on volatile compounds during thermal treatment, and the results showed that the main volatiles produced from PC 18:0/18:1 were nonanal, octanal and (E)-2-decenal, and from PC 18:0/18:2, they were (E, E)-2,4-decadienal, €-2-octenal a€(E)-2-heptenal [12]. Perkins et al. found that the formation of aldehydes was related to the content of linoleic acid and linolenic acid, while alkanes were positively correlated with the content of oleic acid [13]. However, most studies on the relationship between flavor and lipid precursors were limited to the use of lipid standards for their simple oxidation systems. Although the food system is more complex, advancing research would be beneficial for practical applications. Thus, it is worthwhile to conduct in-depth investigations into the relationship between egg yolk’s flavor and lipid precursors.

Oxidized lipids, as intermediate products of lipid oxidation, are crucial in proving the flavor formation mechanism. However, due to their low content and a lack of relevant standards, they are difficult to identify in food [15,16,17]. Oxidized lipids are formed from ordinary lipids through free radical chain reactions and have a strong structural similarity to the molecular structure of lipids. Therefore, the use of structure similarity searching may help in the discovery and characterization of oxidized lipids. Molecular networking (MN) involves pairwise comparisons of the mass spectra in a given dataset, where similar structures tend to produce similar fragmentation patterns. Therefore, the application of the molecular networks in the formation mechanism of egg yolk’s flavor is advantageous for the characterization of flavor intermediates and finding proof of the flavor mechanisms.

Numerous factors can exert a significant impact on the formation of volatile compounds during food processing, with temperature being among the most critical determinants. The temperatures in different cooking methods can change the type and content of volatile compounds by influencing lipid oxidation, leading to the difference in flavor profiles in food [14,18]. Frying or baking food typically involves higher temperatures (140–200 °C) than boiling (100 °C) [19]. Yu et al. investigated the impact of three cooking methods on chicken breast and observed notable variations in volatile flavor compounds. Specifically, phenylacetaldehyde was identified as the primary volatile flavor compound in boiled chicken, 3-butanedione predominated in fried chicken and roasted chicken exhibited the highest levels of 3-methylbutyraldehyde [20]. Similarly, different cooking temperatures can result in varied flavor profiles for egg products, which can impact consumers’ preferences. The consumption of boiled and fried egg products is widespread; however, there is a limited understanding of their flavor profiles and flavor precursors, necessitating further investigation.

**Figure 1:**
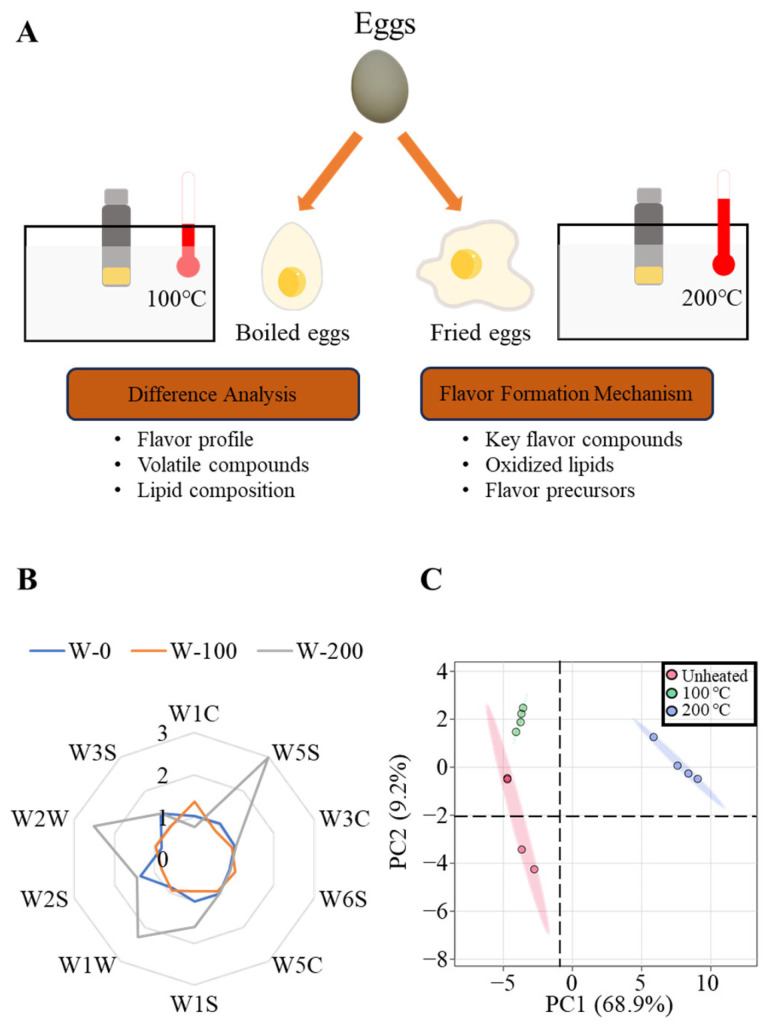
Experimental design and flavor profile analysis of egg yolks. (**A**) Experimental design of the study on the flavor difference of two egg yolk products and flavor formation mechanisms; (**B**) flavor profile analysis using electronic nose; (**C**) PCA of volatile compounds in egg yolks. All assays were performed in triplicate.

In this study, the key flavors of boiled egg yolks (heated at 100 °C, which is a common boiling temperature) and fried egg yolks (heated at 200 °C, which is a common frying temperature) were compared, and their relationship with lipid oxidation was explored (Figure 1A). The flavor profiles, volatile compounds and lipid molecular species were monitored using an electronic nose, gas chromatography–mass spectrometry (GC–MS), gas chromatography–olfactometry–mass spectrometry (GC–O–MS) and ultra-high-performance liquid chromatography–mass spectrometry with a Q-Exactive HF-X Orbitrap (UPLC–Q-Exactive HF-X). The key flavor compounds of boiled egg and fried egg products were discussed by making a comparison of the volatile compounds. Important flavor precursors were also explored with an analysis of the lipids and lipid oxidation products characterized using the molecular network and oxidized lipid molecular weight prediction. This study compared the variability of volatile compounds in different egg products, revealed their key flavor compounds and corresponding lipid precursors and provided a theoretical foundation for the synthesis and development of a flavor essence for heated egg yolks.

## 2. Materials and Methods

### 2.1. Materials

The ammonium formate and cyclohexanone were purchased from Sigma-Aldrich (Madison, WI, USA), while the HPLC-grade solvents, such as methanol, chloroform, isopropanol and acetonitrile, were procured from Spectrum Chemical Mfg., Corp. (Gardena, CA, USA). The 1,2-diheptadecanoyl-sn-glycero-3-phosphoethanolamine (PE; 17:0/17:0), 1,2-diheptadecanoyl-sn-glycero-3-phospho-(1′-sn-glycerol) (PG; 17:0/17:0) and 1,2-diheptadecanoyl-sn-glycero-3-phosphorylcholine (PC; 17:0/17:0) were obtained from Aladdin Reagents Co., Ltd. (Shanghai, China). Additionally, the 1-heptadecanoyl-sn-glycero-3-phosphocholine (LPC; 17:0) was procured from Avanti Polar Lipids (Alabaster, AL, USA), and the triheptadecanoin (TAG; 17:0/17:0/17:0) was sourced from Sigma-Aldrich (Madison, WI, USA).

Fresh eggs were procured from a local market located in Hebi, China. The egg yolks were manually separated from the egg whites after removing the shells. A sample mixture containing at least 10 egg yolks was prepared for each experiment. All the samples were kept on ice until being analyzed to ensure their freshness.

### 2.2. Samples Pretreatment at Different Temperatures

The sample pretreatment for the electronic nose, GC–MS, GC–O–MS and LC–MS analysis was conducted similarly. The egg yolk (2 g) was placed into a 20 mL brown sample bottle. The samples were heated at 100 °C and 200 °C for 10 min using an oil bath, and the bottle was sealed to prevent any volatile loss. Unheated samples served as control samples (Figure 1A). After heating, the samples were quickly cooled on ice for at least 10 min. Then, the samples were immediately further treated for electronic nose, GC–MS, GC–O–MS and UPLC–QE–MS/MS measurement. All assays were performed in triplicate.

### 2.3. Electronic Nose Analysis

The pretreatment samples were incubated for 10 min in a water bath set to 50 °C. A PEN3 electronic nose (WinMuster Airsense Analytics Inc., Schwerin, Germany) was employed to analyze the egg yolk products. To extract volatile gases during the testing process, a hollow needle attached to the tubing was utilized to puncture the vial seal. Subsequently, fresh air, filtered using a charcoal filter, was introduced using a second hollow needle to replace the volatile gases. The duration of each measurement was set at 60 s, with a standby period of 100 s to allow the sensor signals to return to their baseline levels. Then, 10 metal oxide semiconductors were integrated into the electronic nose for selective recognition of diverse volatile classes, and a brief description of each semiconductor used in the study is provided in Table S1.

### 2.4. GC-MS Analysis

The volatile compounds in the pretreatment samples were analyzed using GC–MS (GC7890b, MS5977a MSD, Santa Clara, CA, USA) with an HP-5MS column (30 m × 0.25 mm × 0.25 µm, Agilent Technologies Inc., Palo Alto, CA, USA). To release more volatile compounds, the raw egg yolk in the control sample was covered with the walls of the bottle, while the solidified egg yolk in the heated samples was mashed with a small spoon. Cyclohexanone internal standard (50 µg/mL, 3 μL) was added to the samples, which were then incubated at 60 °C for 20 min. An SPME fiber (PDMS/DVB/CAR) was used to adsorb the volatiles for 40 min at 60 °C. An HP-5MS column was utilized to separate the volatile compounds, and the inlet was set at 250 °C. The GC oven was initially set to 35 °C and maintained for 3 min, followed by an increase of 5 °C/min until it reached 250 °C, and finally held for 10 min. Helium was used as the carrier gas at 1.68 mL/min. The EI source was set at 70 ev. Mass selective detection was conducted in scan mode, scanning from *m*/*z* 50 to 550.

Volatile compounds were identified by comparing their mass spectra with the mass spectra in NIST14. Identification was confirmed using retention index (RI) values, a match factor (similarity > 700) and a reverse match factor (similarity > 700). The linear retention indices of volatiles were calculated using n-Alkanes (C6–C30) [21].

### 2.5. GC–O–MS Analysis

A sniffing port (ODP 3; Gerstel, Mülheim an der Ruhr, Germany) coupled to a GC–MS system with a VF-WAXms column (30 m × 0.25 mm × 0.25 µm) was used for the odor-active volatile compound characterization. The transfer line to the sniffing port was maintained at 240 °C, with the addition of humidified air into the sniffing port at a rate of 60 mL/min. The sample pretreatment was the same as that in the GC–MS detection. A VF-WAXms column was utilized to separate the volatile compounds, and the inlet was set at 250 °C. The GC oven was initially set to 40 °C and maintained for 2 min, followed by an increase of 6 °C/min until it reached 240 °C, and finally held for 5 min. Helium was used as the carrier gas at 1.68 mL/min. The EI source was set at 70 ev. Mass selective detection was conducted in scan mode, scanning from *m*/*z* 50 to 550. The olfactory analysis was performed using three judges. A total of nine GC-O analyses were conducted, with each judge performing three analyses.

### 2.6. Lipidomics Analysis Using UPLC–Q-Exactive HF-X

Lipids in the egg yolk was extracted using the Folch method [22]. Consequently, 0.5 g pretreatment samples were homogenized using 5 mL deionized water. Then, 1.875 mL chloroform/methanol (2:1, *v*/*v*) was added to the samples and shaken, followed by centrifugation at 8000 rpm for 10 min. The organic layers were collected in preweighed vials and dried using a centrifuge concentrator. The dried lipid was then redissolved in methanol as a 10 mg/mL solution. To prepare the samples for UPLC–Q-Exactive HF-X analysis, 30 μL of internal standards (PE 17:0/17:0, PC 17:0/17:0, PG 17:0/17:0, LPC 17:0, TAG 17:0/17:0/17:0) (100 μg/mL), 30 μL of the resolved sample and 240 μL of methanol were combined and vortexed for 6 min to fully dissolve the lipids. Afterward, the samples were purified using centrifugation at 20,000× *g* for 10 min [23] before subjecting them to analysis using UPLC–Q-Exactive HF-X.

The lipid molecular species were analyzed using UPLC–Q-Exactive HF-X (Thermo Fisher, CA, USA). The analytes were separated using an Acquity UPLC BEH C8 column (2.1 mm × 100 mm; 1.7 μm) and an Acquity BEH C8 VanGuard precolumn (2.1 mm × 5 mm; 1.7 μm) (Waters, MA, USA). The mobile phase consisted of acetonitrile/water (6:4, *v*/*v*) (A) and isopropanol/acetonitrile (9:1, *v*/*v*) (B) with 10 mmol/L ammonium formate [23]. The column was maintained at 65 °C and the flow rate was 0.6 mL/min. The following gradient elution was from 0 to 2 min, from 15 to 30% B; from 2 to 2.5 min, from 30 to 48% B; from 2.5 to 11 min, from 48 to 82% B; from 11 to 11.5 min, from 82 to 99% B; from 11.5 to 12 min, 99% B; from 12 to 12.1 min, from 99 to 15% B; from 12.1 to 15 min, 15% B [23].

The MS/MS instrument: The ESI (+)/(−) modes for full MS/ddMS were selected and used. The electrospray ionization source parameters were set as follows: a sweep gas flow rate of 2%, sheath gas flow rate of 60%, auxiliary gas flow rate of 25%, auxiliary gas heater temperature of 370 °C, capillary temperature of 380 °C, positive ion mode spray voltage of 3.60 kV and negative ion mode spray voltage of 3.00 kV.

Non-targeted lipidomics data were utilized to manually annotate the oxidized lipids referring to our previous methods [14]. Firstly, the oxidation products were predicted according to common lipid oxidation mechanisms [14,24]. The *m*/*z* of precursor ions and product ions of the oxidized lipids were calculated in accordance with the lipid fragmentation rules in the Lipidomics Standards Initiative (LSI) guidelines. Then, referring to the calculated precursor ions of the oxidized lipids, we searched for the corresponding *m*/*z* of oxidized lipids in the molecular network. Candidate features with *m*/*z* consistent with the predicted oxidized lipids were further checked for their product ions in the mass spectra using MS-DIAL [25]. Finally, 13 oxidized lipids were identified in the egg yolks.

### 2.7. Data Analysis

The volatile compounds were identified using the MassHunter Workstation Qualitative Analysis software B07.00 (Agilent Technologies). Statistical significance analysis was performed on both the volatile compounds and lipids data using SPSS 26.0 (SPSS Inc., Chicago, IL, USA). Differences between samples were assessed for statistical significance using the one-way ANOVA test with a significance level set at *p* < 0.05. The lipid data were processed using MS-DIAL [26], and then subjected to principal component analysis (PCA), Partial Least Squares Discriminant Analysis (PLS-DA) and Variable Importance in Projection (VIP) using MetaboAnalyst 4.0 [27]. To enhance the readability due to the abundance of data points in the loading plot, we performed additional processing on the loading plot data in Microsoft Excel 2016 (Microsoft Excel, Redmond, DC, USA). The VIP data exported from MetaboAnalyst 4.0 were also reprocessed in Microsoft Excel. The nodes and edges of the molecular network were exported from MS-DIAL and the molecular network was constructed using Cytoscape v3.2 [28]. OmicStudio was utilized for the correlation heatmap between the volatile compounds and lipid molecular species [29].

## 3. Results and Discussions

### 3.1. Flavor Profile Analysis of Egg Yolks

The flavor profiles of the egg yolks at varying temperatures (they simulated boiled egg yolks and fried egg yolks, respectively) are shown in Figure 1. The 10 sensors of the electronic nose are sensitive to different volatile compounds (Table S1). Figure 1B shows the corresponding differences in the electronic noses of the different egg yolk products. The value in the radar chart represents the content of the corresponding flavor compounds. Comparing the electronic nose data of egg yolks across different temperatures (Figure 1B), the flavor profile of 200 °C-heated yolks showed the most significant difference. The responses of W5S, W1S, W1W and W2W in the 200 °C-heated yolks exhibited significantly higher levels compared to both the unheated yolks and the 100 °C-heated yolks, indicating that the content of nitrogen oxides, methane, sulfur compounds, pyrazine, terpenes, aromatic components and sulfur-containing compounds in the 200 °C-heated yolks may be higher than in the other two samples. The 100 °C-heated yolks had a higher response at W1C alone than the other two samples, suggesting that boiled eggs contained more aromatic compounds. The flavor profile of raw yolks was similar to the 100 °C-heated yolks, but slightly higher at W1S, W2S, W3S and W5S than the latter, indicating the raw yolks contained more nitrogen oxides, methane, ethanol and aromatic compounds, etc., than the 100 °C-heated yolks.

Principal component analysis (PCA) was applied to the GC–MS data of the egg yolks (Figure 1C). The total variance contribution of the first two principal components was 77.9%, which could represent the information of most volatile compounds. Different samples (including the raw yolks, 100 °C-heated yolks and 200 °C-heated yolks) were completely distinguished, suggesting that their flavor profiles had significant differences.

### 3.2. Change in Volatile Flavor Compounds of Egg Yolks during Thermal Treatment

**Figure 2:**
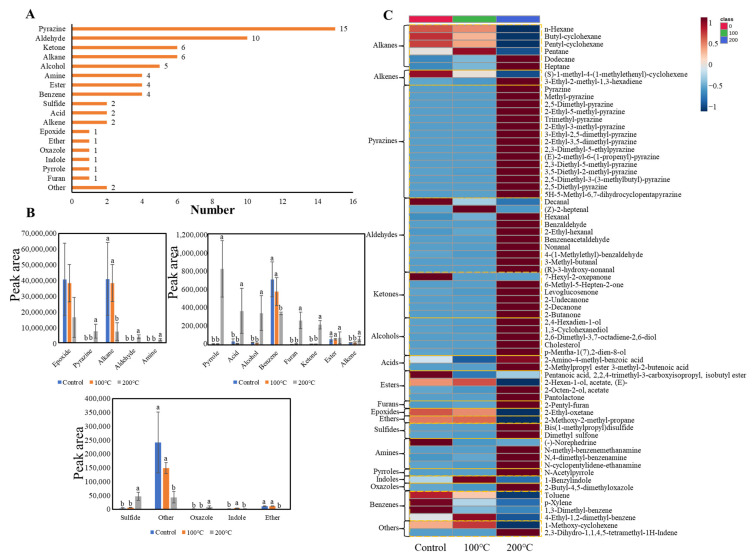
Volatile compound changes in egg yolks during thermal treatment. (**A**) Category comparison of volatile compounds; (**B**) changes in volatile compounds of different categories during thermal treatment; (**C**) heatmap analysis of volatile compounds during thermal treatment. Values of different groups with different lower-case letters (a,b) are significantly different at *p* < 0.05. All assays were performed in triplicate.

The volatile compounds of egg yolks heated at different temperatures were analyzed using GC–MS and GC–O–MS equipped with non-polar and polar columns, respectively. A total of 68 volatile compounds were identified using multiple screening based on matching scores, mass spectral characteristic ions and retention index (RI) values (Table S2). The majority of the volatile compounds (58) were found to contain oxygen and nitrogen. Among them, pyrazines (15), aldehydes (10), ketones (6) and alcohols (5) were identified most frequently. Additionally, two sulfur compounds, two alkenes and six alkanes were identified (Figure 2A). Figure 2B shows the changes in different categories of volatile compounds during thermal treatment. Epoxy compounds, alkanes, benzene compounds and ethers showed higher levels in the raw and boiled eggs. Pyrazines, aldehydes, amine compounds, pyrroles, acids, alcohols, furans, ketones, alkenes, sulfur compounds and oxazoles exhibited higher levels in the fried eggs, indicating that these compounds were formed during high-temperature heating. Indole compounds showed higher levels in the boiled eggs, suggesting that they might be characteristic volatile compounds formed at 100 °C. Figure 2C illustrates the changes in volatiles at different temperatures. In the heatmap, the colors range from a cool color (e.g., blue) to a warm color (e.g., red), representing low and high values, respectively. All pyrazines increased with an increasing temperature, indicating that they were easier to form at high temperatures. Pyrazines are also found in many baked foods that need high-temperature processing [30,31]. All alcohols, acids, furans, as well as most aldehydes, ketones and amine compounds increased with an increasing heating temperature. The formation of numerous oxygen and nitrogen-containing compounds during high-temperature heating suggests their close relationship with the Maillard reaction and lipid oxidation. In the 200 °C samples, there were 49 compounds with relatively higher levels, of which 39 had carbon chains of 6–10 carbons. Among them, 11 oxygen-containing compounds with carbon chains of 8–10 carbons might be related to the oxidation of oleic acid [14].

### 3.3. Analysis of Key Volatile Flavor Compounds of Egg Yolks via GC–O–MS

**Figure 3:**
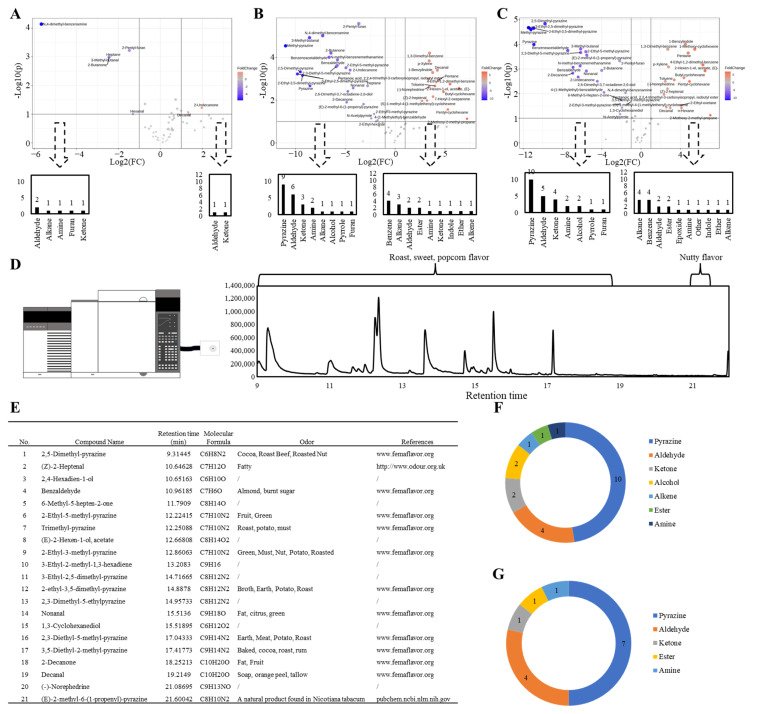
Analysis of key volatiles of egg yolks during thermal treatment. (**A**) Volcano plot between raw egg yolks and boiled egg yolks; (**B**) volcano plot between raw egg yolks and fried egg yolks; (**C**) volcano plot between boiled egg yolks and fried egg yolks; (**D**) chromatogram of key flavor volatile compounds and flavor description during GC–O–MS analysis; (**E**) the volatile compounds identified during the period with a strong/significant flavor in GC–O–MS analysis; (**F**) category statistics for volatile compounds identified within 9–23 min in fried eggs; (**G**) category statistics for volatile compounds identified within 9–23 min and significantly increased in fried eggs. Compared to raw eggs, the contents of compounds numbered 1, 2, 4, 6, 8, 9, 11, 12, 14, 16, 18, 19, 20, and 21 in subfigure (**E**) were found to significantly increase in fried eggs. These compounds were classified and plotted in subfigure (**F**). All assays were performed in triplicate.

In order to explore the key flavor compounds in different egg yolk products, we detected the egg yolk samples using GC–O–MS and analyzed the difference in compound content and its retention time in the chromatographic column. Figure 3A–C demonstrate the key differential volatiles in different egg yolk samples. In the volcano plot, significant features are depicted as points that fall outside the “volcano” shape, with points that are farther away from the center indicating higher significance and larger effect sizes. As shown in Figure 3A, six compounds were significantly elevated in the boiled eggs. Hexanal exhibited a grassy, tallow and fatty flavor (www.femaflavor.org (accessed on 1 June 2023)), 2-pentyl-furan presented a buttery, floral, fruity and green bean flavor (www.femaflavor.org (accessed on 1 June 2023)), heptane showed an alkane, ethereal and sweet flavor (https://cosylab.iiitd.edu.in/ (accessed on 1 June 2023)), 3-methyl-butanal exhibited an almond, cheese, chocolate and malt flavor (http://www.odour.org.uk (accessed on 1 June 2023)) and 2-butanone displayed a fragrant, fruity and pleasant flavor (www.femaflavor.org (accessed on 1 June 2023)). The flavor of N,4-dimethyl-benzenamine was not clear. Due to the mild flavor of the boiled eggs, the experimenters did not detect any related aromas in the GC–O–MS analysis. However, based on the flavor descriptions of the above compounds, it can be inferred that the first five compounds may contribute significantly to the oily and cheesy flavors of boiled egg yolks. During the thermal treatment at 100 °C, four oxygen-containing compounds, one nitrogen-containing compound and one alkane were formed, and the oxygen-containing compound content far exceeded that of other types of volatile compounds. This indicated that lipid oxidation contributed a lot to the boiled egg yolk flavor. Figure 3B shows a volcano plot of the volatile compounds in fried and raw eggs. A large number of volatile compounds were formed in the fried eggs, which differed significantly from those in the raw eggs. Oxygen- and nitrogen-containing compounds were formed in large quantities during the thermal treatment at 200 °C, including nine pyrazines, two amines, one pyrrole, six aldehydes, three ketones, one alcohol and one furan. The differences in volatile compounds between the fried and boiled eggs were similar to those between the fried and raw eggs (Figure 3C). There are many volatile compounds significantly increased in fried eggs and thus the key flavor volatile compounds in fried eggs need to be further determined using GC–O–MS. The experimenters smelled the fried egg samples during GC–O–MS analysis and detected several distinct flavors between 9 and 23 min, with the main flavor descriptors being roasted, sweet, popcorn and nutty flavors (Figure 3D). The volatile compounds formed during this time are shown in Figure 3E. Ultimately, 21 volatile compounds were identified during this period, with pyrazines and aldehydes being the majority (Figure 3F). Combining the analysis results in the difference between fried and raw eggs (Figure 3B), 14 volatile compounds, with retention times between 9 and 23 min, were identified as significantly higher in fried eggs than in raw eggs, including eight nitrogen-containing compounds and six oxygen-containing compounds (Figure 3G). Among these volatile compounds, 11 possessed associated flavor descriptors (Figure 3E) and made a significant contribution to the flavor of the fried eggs. Particularly from 12.8 to 17.5 min, a strong sweet and roasted aroma was detected during GC–O–MS, corresponding to the volatile compounds 2-ethyl-3-methyl-pyrazine, 3-ethyl-2,5-dimethyl-pyrazine, 2-ethyl-3,5-dimethyl-pyrazine, nonanal and 2,3-diethyl-5-methyl-pyrazine. Therefore, the flavor of fried eggs mainly came from 14 volatile compounds, with the most critical ones being 2-ethyl-3-methyl-pyrazine, 3-ethyl-2,5-dimethyl-pyrazine, 2-ethyl-3,5-dimethyl-pyrazine, nonanal and 2,3-diethyl-5-methyl-pyrazine.

### 3.4. Lipid Molecular Species Profile Analysis of Egg Yolks

**Figure 4:**
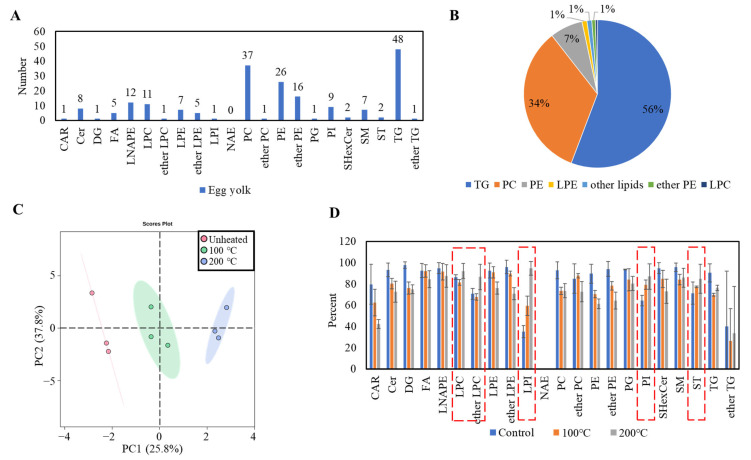
Lipid composition changes in egg yolks during thermal treatment. (**A**) The types and quantities of lipid molecules in egg yolks; (**B**) the comparison of the content of different categories of lipid molecules in egg yolks; (**C**) PLS-DA of lipid molecules in egg yolks; (**D**) content changes in different subclasses of lipids in egg yolks during thermal treatment. Lipids on the rise were marked in the red dotted box.

A total of 201 lipid molecules were identified in the egg yolks (Table S3), belonging to 21 lipid subclasses (Figure 4A). The TG (triglyceride) and PC (phosphatidylcholine) contents in the egg yolks were the highest (Figure 4B). PLS-DA was employed to analyze the lipid molecular species data in Figure 4C, and their total variance accounted for 63.6%. The score plot made a significant distinction among the egg yolks heated at different temperatures, indicating the lipid composition of egg yolks possessed marked changes during the heating treatment.

To explore the content changes in different types of lipids during heating treatment, the lipidomic data were categorized and analyzed in Figure 4D. The elevated lipids primarily included LPC (lysophosphatidylcholine), ether LPC, LPI (lysophosphatidylinositol), PI (phosphatidylinositol) and ST (sphingophospholipid), while most other types of lipids exhibited a declining trend. LPC, ether LPC and LPI belong to the lysophospholipids that could be generated by the ester bond cleavage reaction of phospholipids during lipid oxidation [14]. On the other hand, although LPE (lysophoshatidylethanolamine) also belongs to lysophospholipids, it could further degrade into other oxidation products due to its poor oxidation stability [14]. The lipids with higher contents in the egg yolks, including TGs, PCs, PEs (phosphoethanolamines), ether PEs and LPEs, exhibited significant changes during the heating treatment, indicating their potential contribution to their flavor formation.

### 3.5. Exploration of Key Lipids Associated with the Flavor of Egg Yolks

**Figure 5:**
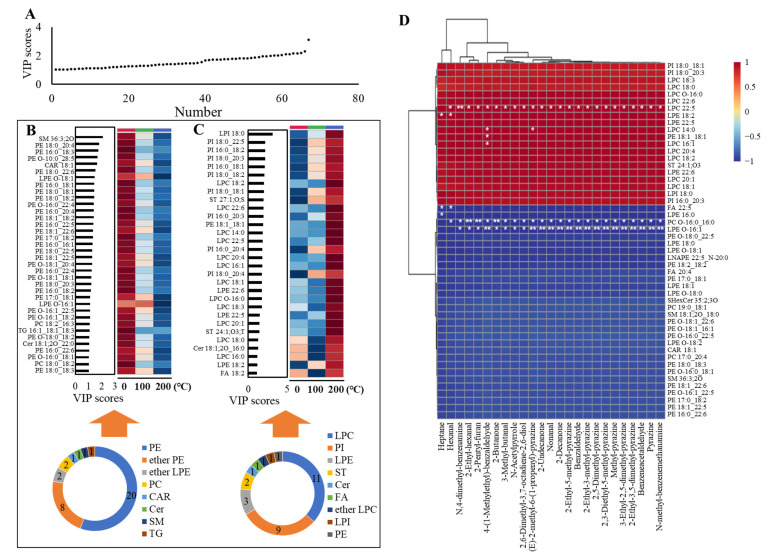
Analysis of key lipid precursors of egg yolk flavor compounds. (**A**) 67 lipids with VIP > 1 in egg yolks; (**B**) analysis of the type and amount of lipids with a decreasing trend in egg yolks; (**C**) analysis of the type and amount of lipids with an increasing trend in egg yolks; (**D**) inter-group correlation between volatile compounds and lipid molecular species in egg yolks. * < 0.05, ** < 0.01.

To elucidate the key flavor precursors in egg yolk products, VIP analysis and inter-group correlation analysis were employed to analyze the lipid molecular species data. The two analytical methods focused on the importance of variables and the correlation between changing trends in lipids and changing trends in key volatile compounds, respectively, and their comprehensive analysis increased the reliability of the flavor precursor inference. During VIP analysis, 67 lipids with VIP values exceeding 1 were identified in the egg yolks (Figure 5A). Among the egg yolk samples, 36 lipids (VIP > 1) displayed a decreasing tendency, encompassing 20 PEs and 8 ether PEs (Figure 5B), suggesting that the oxidative degradation of these phospholipids in egg yolks may be an important source of its flavor. On the other hand, 31 lipids demonstrated an increasing trend, predominantly LPCs, PIs and LPEs (Figure 5C). The significant formation of lysophospholipids may originate from the oxidative degradation of PC and PE. Although PCs had no significant decrease in the VIP analysis, this may be due to their high content and large base. The increase in PIs suggested that they may result from other phospholipids binding with inositol during heating treatment. Both types of phospholipids can form oxygen-containing volatiles due to thermal oxidation, especially aldehydes [14]. Therefore, the egg yolk flavor after heating treatment may come from phospholipids, especially PE-related lipids.

An inter-group correlation analysis between the volatile compounds and lipids was performed to further clarify the flavor precursors. Consequently, 25 volatile compounds filtered using a volcano plot and the 50 lipids most correlated with these volatiles were selected and analyzed, as shown in Figure 5D. The lipids that exhibited negative correlations with the volatile compounds are considered potential precursors of these volatile compounds. The majority of the key flavor volatile compounds in the boiled egg yolks and fried egg yolks exhibited negative correlations with PC O-16:0_16:0 and LPE O-16:1. Heptane demonstrated negative correlations with FA 22:5 and LPE 16:0. Hexanal showed negative correlations with FA 22:5 and PC O-16:0_16:0. Hence, the flavors of boiled egg yolks and fried egg yolks may have an important relationship with the oxidation of phospholipids and FAs. Hence, combining the VIP analysis results, the key flavor precursors in boiled eggs and fried egg yolks may primarily be derived from phospholipids and FAs, especially PE-related lipids.

### 3.6. Characterization and Analysis of Oxidized Lipids in Egg Yolk Combined with the Molecular Network

**Figure 6:**
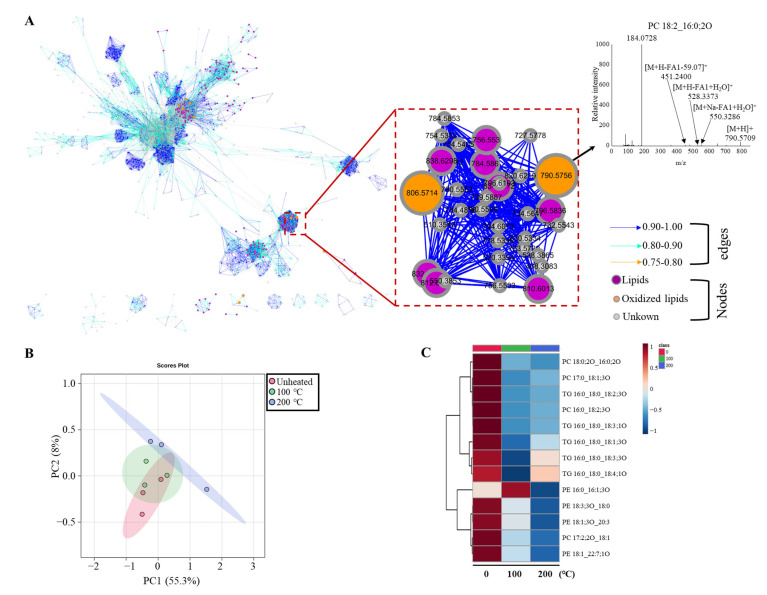
Characterization and analysis of key oxidized lipids in egg yolks using molecular networks. (**A**) Characterization of oxidized lipids via molecular networks; (**B**) PLS-DA of oxidized lipids in egg yolks; (**C**) heatmap of oxidized lipids in egg yolks. All assays were performed in triplicate.

Oxidized lipids are important intermediates during flavor formation. Currently, there is no large-scale characterization method for oxidized lipids [32]. Molecular networking is based on the principle that compounds with similar structures form similar fragmentation patterns in mass spectra to establish connections between different compounds [33]. This approach is useful for discovering oxidized lipids. Therefore, this study used a molecular network combined with the predicted *m*/*z* of oxidized lipids to characterize the oxidized lipids in egg yolks. The 100 lipids with larger VIP values in the egg yolk were screened, and the *m*/*z* of their oxidized lipids were predicted. Meanwhile, as shown in Figure 6A, the feature dots with similar mass spectra were clustered using a molecular network to narrow down the search range. The feature dots with the same *m*/*z* as the predicted oxidized lipids were selected in the molecular network. Finally, the oxidized lipids in the egg yolks were determined by checking the diagnostic ions (including lipid class-specific fragments and lipid molecular species fragments) in the mass spectra of the candidate feature dots in accordance with the lipid fragmentation rules in the Lipidomics Standards Initiative (LSI) guidelines.

Thirteen oxidized lipids were identified in the egg yolks, as shown in Figure 6. The 200 °C-heated yolks and the raw yolks could be completely separated using PLS-DA, but the 100 °C-heated yolks could not (Figure 6B). This indicated that the oxidized lipids in the fried eggs underwent significant changes. The heatmap shows the change in the oxidized lipids with temperature (Figure 6C). Most of the oxidized PLs in both types of egg yolks showed a downward trend, indicating that they may have originally existed in the egg yolks but were not generated by lipid oxidation. This may be because the amount of oxidized PLs generated during the brief heating treatment was small and not detectable, while the ones originally present in egg yolks had a larger content that was easier to detect. In addition, PE 16:0_16:1;3O was one of the few intermediate products formed during the heating treatment in the egg yolks, exhibiting a trend of an initial increase followed by a decrease. It was an oxidized lipid formed during the heating treatment, belonging to the phosphoethanolamines. This also proved that PE-related lipids may serve as prominent flavor precursors in fried yolk flavor formation. This result was also in agreement with the analytical results in the previous section (Figure 5).

Phospholipids have been shown to impact the formation of flavor in animal-based foods through lipid oxidation reactions. Chen et al. found that the addition of neutral lipids had little effect on the flavor of chicken, but the addition of egg yolk phospholipids could increase the aroma of chicken and increase the concentration of most lipid-derived volatiles, such as the two Strecker aldehydes (2- and 3-methylbutanal) [34]. This provided evidence for the formation of aldehydes through phospholipid oxidation reactions, and also proved that 3-methyl-butyral, as a key flavor substance in boiled eggs, can be produced by yolk phospholipids (Figure 3A). Numerous studies have reported PEs to be a precursor of food flavor. Li et al. identified a significant decline in the PE levels in heated sturgeon meat, which exhibited a strong correlation with the production of various aldehydes [35]. The significantly increased aldehydes in fried eggs included (Z)-2-heptenal, benzaldehyde, nonanal and decanal. The formation of nonanal and decanal is associated with the degradation of oleic acid [14,34], so they may be generated from the large number of PEs with 18-carbon fatty acids in Figure 5B. Furthermore, the interaction between lipids and the Maillard reaction is a crucial pathway for the development of egg yolk’s flavor. Zhao et al. discovered that lipid oxidation generated a substantial number of free radicals, precursors of advanced glycation end products (AGEs) and highly reactive glycation compounds. These substances can react with amino acids or amino groups, thereby enhancing the formation of Maillard reaction products [36]. Francisco et al. found that benzaldehyde could be formed through the amino acid degradation pathways initiated by lipid oxidation products [37]. Benzaldehyde also made an important contribution to the flavor of fried egg yolks in this study, so it may be also produced through a complex lipid oxidation and Maillard reaction. Pyrazines are important products of the Maillard reaction. They played the most important role in the flavor of egg yolks, giving them a roasted and sweet aroma. Their generation may also be closely related to lipid oxidation [38]. Negroni, D’Agostin and Arnoldi evaluated the influence of three edible oils with different unsaturation levels on lysine–glucose and lysine–xylose models. Their results suggested that while the total content of unsubstituted pyrazines decreased with increasing unsaturation levels of lipids, the formation of substituted pyrazines was promoted. The study conducted by Yu et al. revealed that highly unsaturated lipids play a crucial role in promoting the synthesis of pyrazine compounds with long side chains, such as 2,3-diethyl-5-methylpyrazine and 3,5-diethyl-2-methylpyrazine. Conversely, lipids with low unsaturation levels were found to facilitate the formation of pyrazine compounds with shorter side chains, such as 2,6-dimethylpyrazine and 2,5-dimethylpyrazine [39]. In this study, the four pyrazines that contributed the most to the egg yolk flavor were identified as 2-ethyl-3-methyl-pyrazine, 3-ethyl-2,5-dimethyl-pyrazine, 2-ethyl-3,5-dimethyl-pyrazine and 2,3-diethyl-5-methyl-pyrazine, all of which belonged to the class of pyrazines with long side chains. This further suggests a close relationship between the reduction of PEs containing polyunsaturated fatty acids and the formation of pyrazine compounds during thermal treatment. On the other hand, some studies have also shown that the amino group of PEs may directly undergo a Maillard reaction with the carbonyl group of reducing sugar during thermal treatment [40,41,42]. Therefore, PEs containing polyunsaturated fatty acid chains played a significant role in the flavor development of egg yolks, including the production of aldehydes and pyrazine compounds, through lipid oxidation reactions and the Maillard reaction. However, there is currently no definite evidence that PEs directly participate in the Maillard reaction to form pyrazine compounds. The relationship between PEs and pyrazine compounds needs further study.

## 4. Conclusions

In conclusion, the comprehensive lipidomics and flavoromics analysis uncovered the flavor profiles, key flavor compounds and flavor precursors of egg yolks at different temperatures. The flavor of the boiled eggs originated from hexanal, 2-pentyl-furan, 2-butanone, 3-methyl-butanal and heptane, most of which were oxygen-containing compounds mainly formed by lipid oxidation. The key volatile compounds in the fried eggs were 2-ethyl-3-methyl-pyrazine, 3-ethyl-2,5-dimethyl-pyrazine, 2-ethyl-3,5-dimethyl-pyrazine, nonanal and 2,3-diethyl-5-methyl-pyrazine, providing a baked and burnt sugar flavor. Due to the relatively short heating time, the thermal treatment possessed little impact on the nutritional value of lipids, and only a small portion of the lipid content changed, contributing to the flavor of the egg yolks. PEs containing polyunsaturated fatty acids were the primary flavor precursors contributing to the development of the egg yolk’s flavor. They participated in lipid oxidation reactions and the Maillard reaction, regulating the production of aldehydes and pyrazine compounds. This study revealed the key flavor compounds and their precursors in egg yolk products, providing reference and guidance for the development of and controlled release research on egg yolk flavor products.

## Data Availability

The data presented in this study are available in the article and Supplementary Materials.

## Supplementary Materials

The following supporting information can be downloaded at: https://www.mdpi.com/article/10.3390/foods13020226/s1, Table S1: 10 electronic nose sensors and their corresponding compound types.; Table S2: Retention Time, Compound Name, Matching Scores, Reverse Matching Scores, Molecular Formula, Detection Method of 68 volatile compounds generated from egg yolks during thermal treatment; Table S3: Compound name, average *m*/*z*, average retention time (min), formula, adduct type of lipid molecular species in egg yolks.
