# Supplementary material for: Unraveling the Formation Mechanism of Egg’s Unique Flavor via Flavoromics and Lipidomics
Source: Foods. 2024 Jan 10;13(2):226. doi: 10.3390/foods13020226 (PMC10814943; doi:10.3390/foods13020226)
Supplement: Supplementary file 1 [file foods-13-00226-s001.zip › foods-2797405-supplementary.pdf]

Supporting information for:

Unraveling the Formation Mechanism of Egg Unique Flavor via Flavoromics and Lipidomics

Zheng Zhou, Shuang Cui, Jing Che, Yu-Ying Zhang, Dayong Zhou, Xuhui Huang*, Lei Qin*

State Key Laboratory of Marine Food Processing & Safety Control, School of Food Science and Technology, National Engineering Research Center of Seafood, Dalian Polytechnic University, Dalian 116034, China

***Corresponding Author**

Xuhui Huang

E-mail: xuhuisos@dlpu.edu.cn

Lei Qin

Phone: +86-411-86323262

E-mail: qinlei@dlpu.edu.cn

Table S1. 10 electronic nose sensors and their corresponding compound types.

Semiconductors	Compounds
W1C	aromatic compounds
W1S	methane, broad range of compounds
W1W	sulfur compounds, terpenes
W2S	broad range, alcohols
W2W	aromatics and organic sulfur compounds
W3C	ammonia, aromatic compounds
W3S	methane and aliphatic compounds
W5C	alkanes and aromatics
W5S	broad range
W6S	hydrocarbons

Table S2. Retention Time, Compound Name, Matching Scores, Reverse Matching Scores, Molecular Formula, Detection Method of 68 volatile compounds generated from egg yolks during thermal treatment.

No.	Retention Time (min)	Compound Name	Matching Scores	Reverse Matching Scores	Molecular Formula	Detection Method
1	2.3237	n-Hexane	969	971	C6H14	GC-MS
2	2.3237	2-Ethyl-oxetane	816	921	C5H10O	GC-MS
3	4.489917	Pyrazine	697	797	C4H4N2	GC-MS
4	5.046183	Toluene	704	790	C7H8	GC-MS
5	5.923367	Hexanal	942	954	C6H12O	GC-MS+GC-O-MS
6	6.757767	Methyl-pyrazine	859	890	C5H6N2	GC-MS+GC-O-MS
7	7.982617	p-Xylene	707	818	C8H10	GC-MS
8	7.982617	1,3-Dimethyl-benzene	703	825	C8H10	GC-MS
9	9.282367	1-Methoxy-cyclohexene	863	875	C7H12O	GC-MS
10	9.31445	2,5-Dimethyl-pyrazine	924	953	C6H8N2	GC-MS
11	9.421417	2-Amino-4-methyl-benzoic acid	549	549	C8H9NO2	GC-MS
12	10.64628	(Z)-2-heptenal	661	794	C7H12O	GC-MS
13	10.65163	2,4-Hexadien-1-ol	599	709	C6H10O	GC-MS
14	10.91907	N-Acetylpyrrole	939	939	C6H7NO	GC-MS
15	10.96185	Benzaldehyde	887	887	C7H6O	GC-MS
16	11.64113	2-Ethyl-hexanal	667	725	C8H16O	GC-MS
17	11.7909	6-Methyl-5-Hepten-2-one	527	715	C8H14O	GC-MS
18	11.9674	2-Pentyl-furan	818	901	C9H14O	GC-MS
19	12.22415	2-Ethyl-5-methyl-pyrazine	648	648	C7H10N2	GC-MS
20	12.25088	Trimethyl-pyrazine	909	909	C7H10N2	GC-MS+GC-O-MS
21	12.66808	2-Hexen-1-ol, acetate, (E)-	616	649	C8H14O2	GC-MS
22	12.86063	2-Ethyl-3-methyl-pyrazine	635	734	C7H10N2	GC-MS
23	13.09598	(S)-1-methyl-4-(1-methylethenyl)-cyclohexene	693	831	C10H16	GC-MS
24	13.19227	Butyl-cyclohexane	744	809	C10H20	GC-MS

25	13.2083	3-Ethyl-2-methyl-1,3-hexadiene	556	638	C9H16	GC-MS
26	13.63085	Benzeneacetaldehyde	751	753	C8H8O	GC-MS+GC-O-MS
27	13.91968	Levoglucosenone	557	619	C6H6O3	GC-MS
28	13.63085	N-methyl-benzenemethanamine	579	650	C8H11N	GC-MS
29	14.21387	2-Methylpropyl ester 3-methyl-2-butenoic acid	579	736	C9H16O2	GC-MS
30	14.0427	2-Butyl-4,5-dimethyloxazole	567	567	C9H15NO	GC-MS
31	14.71665	3-Ethyl-2,5-dimethyl-pyrazine	953	959	C8H12N2	GC-MS
32	14.8878	2-Ethyl-3,5-dimethyl-pyrazine	789	803	C8H12N2	GC-MS
33	14.8878	4-Ethyl-1,2-dimethyl-benzene	544	583	C10H14	GC-MS
34	14.95733	2,3-Dimethyl-5-ethylpyrazine	881	928	C8H12N2	GC-MS
35	15.17128	2-Undecanone	848	963	C11H22O	GC-MS
36	15.51895	1,3-Cyclohexanediol	535	648	C6H12O2	GC-MS
37	15.36383	(E)-2-methyl-6-(1-propenyl)-pyrazine	806	806	C8H10N2	GC-MS
38	15.5136	Nonanal	934	934	C9H18O	GC-MS
39	16.27847	N,4-dimethyl-benzenamine	577	720	C8H11N	GC-MS
40	16.46567	Pentyl-cyclohexane	698	799	C11H22	GC-MS
41	16.58868	5H-5-Methyl-6,7-dihydrocyclopentapyrazine	918	918	C8H10N2	GC-MS
42	16.77055	N-cyclopentylidene-ethanamine	816	897	C7H13N	GC-MS
43	17.04333	2,3-Diethyl-5-methyl-pyrazine	741	801	C9H14N2	GC-MS
44	17.41773	3,5-Diethyl-2-methyl-pyrazine	730	850	C9H14N2	GC-MS
45	18.25213	2-Decanone	583	784	C10H20O	GC-MS
46	18.34842	Dodecane	686	788	C12H26	GC-MS
47	18.34842	2-Octen-2-ol, acetate	660	824	C10H18O2	GC-MS
48	18.5891	2,6-Dimethyl-3,7-octadiene-2,6-diol	566	611	C10H18O2	GC-MS
49	19.0277	4-(1-Methylethyl)-benzaldehyde	631	683	C10H12O	GC-MS
50	19.2149	Decanal	550	603	C10H20O	GC-MS
51	21.08695	(-)-Norephedrine	533	596	C9H13NO	GC-MS
52	21.60042	2,5-Dimethyl-3-(3-methylbutyl)-pyrazine	692	734	C11H18N2	GC-MS
53	22.67552	2,3-Dihydro-1,1,4,5-tetramethyl-1H-Indene	618	695	C13H18	GC-MS
54	28.58583	Pentanoic acid, 2,2,4-trimethyl-3-carboxyisopropyl, isobutyl ester	585	688	C16H30O4	GC-MS
55	47.3063	Cholesterol	926	930	C27H46O	GC-MS

56	2.25	Pentane	902	902	C5H12	GC-O-MS
57	2.436	2-Methoxy-2-methyl-propane	865	876	C5H12O	GC-O-MS
58	2.488	Heptane	929	929	C7H16	GC-O-MS
59	2.85	2-Butanone	876	920	C4H8O	GC-O-MS
60	3.578	3-Methyl-butanal	910	914	C5H10O	GC-O-MS
61	6.8	1-Benzylindole	714	714	C15H13N	GC-O-MS
62	10.566	(R)-3-hydroxy-nonanal	753	962	C9H18O2	GC-O-MS
63	14.844	Bis(1-methylpropyl)disulfide	511	648	C8H18S2	GC-O-MS
64	15.51	2,5-Diethyl-pyrazine	478	665	C8H12N2	GC-O-MS
65	22.503	Dimethyl sulfone	706	910	C2H6O2S	GC-O-MS
66	22.7	p-Mentha-1(7),2-dien-8-ol	424	774	C10H16O	GC-O-MS
67	24.56	Pantolactone	924	947	C6H10O3	GC-O-MS
68	27.628	7-Hexyl-2-oxepanone	509	606	C12H22O2	GC-O-MS

Table S3. Compund name, average m/z, average retention time (min), formula, adduct type of lipid molecular species in egg yolks.

No.	Metabolite name	Average m/z	Average Rt(min)	Formula	Adduct type
1	CAR 18:1	426.3578	1.633	$C_{25}H_{48}NO_4$	$[M]^+$
2	Cer 18:1;2O/16:0	536.5063	5.847	$C_{34}H_{67}NO_3$	$[M-H]^-$
3	Cer 18:1;2O/22:0	620.5989	7.932	$C_{40}H_{79}NO_3$	$[M-H]^-$
4	Cer 18:1;2O/22:0;(2OH)	636.5947	7.605	$C_{40}H_{79}NO_4$	$[M-H]^-$
5	Cer 18:1;2O/23:0	634.6143	8.257	$C_{41}H_{81}NO_3$	$[M-H]^-$
6	Cer 18:1;2O/24:0	648.6301	8.57	$C_{42}H_{83}NO_3$	$[M-H]^-$
7	Cer 18:1;2O/24:0;(2OH)	664.6264	8.259	$C_{42}H_{83}NO_4$	$[M-H]^-$
8	Cer 18:1;2O/24:1	646.6134	7.902	$C_{42}H_{81}NO_3$	$[M-H]^-$
9	DG 18:1_18:2	636.5565	6.655	$C_{39}H_{70}O_5$	$[M+NH4]^+$
10	FA 18:1	281.2492	3.209	$C_{18}H_{34}O_2$	$[M-H]^-$
11	FA 18:2	279.2324	5.285	$C_{18}H_{32}O_2$	$[M-H]^-$
12	FA 20:4	303.234	2.479	$C_{20}H_{32}O_2$	$[M-H]^-$
13	FA 22:5	329.2485	2.842	$C_{22}H_{34}O_2$	$[M-H]^-$
14	FA 22:6	327.2328	2.262	$C_{22}H_{32}O_2$	$[M-H]^-$
15	LNAPE 16:0/N-18:0	718.5391	5.716	$C_{39}H_{78}NO_8P$	$[M-H]^-$
16	LNAPE 18:1/N-18:0	744.5537	5.787	$C_{41}H_{80}NO_8P$	$[M-H]^-$
17	LNAPE 18:2/N-18:0	742.5411	5.285	$C_{41}H_{78}NO_8P$	$[M-H]^-$
18	LNAPE 18:1/N-20:0	772.59	6.463	$C_{43}H_{84}NO_8P$	$[M-H]^-$
19	LNAPE 18:2/N-20:0	770.5706	5.944	$C_{43}H_{82}NO_8P$	$[M-H]^-$
20	LNAPE 18:1/N-20:2	768.5582	5.355	$C_{43}H_{80}NO_8P$	$[M-H]^-$
21	LNAPE 20:4/N-18:0	766.5388	5.187	$C_{43}H_{78}NO_8P$	$[M-H]^-$
22	LNAPE 20:4/N-20:0	794.5746	5.839	$C_{45}H_{82}NO_8P$	$[M-H]^-$
23	LNAPE 20:4/N-20:1	792.5589	5.236	$C_{45}H_{80}NO_8P$	$[M-H]^-$
24	LNAPE 22:5/N-18:0	792.5579	5.442	$C_{45}H_{80}NO_8P$	$[M-H]^-$
25	LNAPE 22:6/N-18:0	790.5385	5.02	$C_{45}H_{78}NO_8P$	$[M-H]^-$
26	LNAPE 22:5/N-20:0	820.5878	6.094	$C_{47}H_{84}NO_8P$	$[M-H]^-$
27	LPC 14:0	468.308	0.961	$C_{22}H_{46}NO_7P$	$[M+H]^+$
28	LPC 16:0	496.3392	1.499	$C_{24}H_{50}NO_7P$	$[M+H]^+$
29	LPC 16:1	494.3243	1.073	$C_{24}H_{48}NO_7P$	$[M+H]^+$
30	LPC 18:0	524.3702	2.323	$C_{26}H_{54}NO_7P$	$[M+H]^+$
31	LPC 18:1	522.3554	1.662	$C_{26}H_{52}NO_7P$	$[M+H]^+$
32	LPC 18:2	520.3396	1.23	$C_{26}H_{50}NO_7P$	$[M+H]^+$
33	LPC 18:3	518.3256	0.997	$C_{26}H_{48}NO_7P$	$[M+H]^+$
34	LPC 20:1	550.3889	2.473	$C_{28}H_{56}NO_7P$	$[M+H]^+$
35	LPC 20:4	544.3396	1.201	$C_{28}H_{50}NO_7P$	$[M+H]^+$
36	LPC 22:5	570.3541	1.479	$C_{30}H_{52}NO_7P$	$[M+H]^+$
37	LPC 22:6	568.3415	1.147	$C_{30}H_{50}NO_7P$	$[M+H]^+$
38	LPC O-16:0	482.3604	1.781	$C_{24}H_{52}NO_6P$	$[M+H]^+$
39	LPE 16:0	452.2784	1.527	$C_{21}H_{44}NO_7P$	$[M-H]^-$
40	LPE 18:0	480.3106	2.376	$C_{23}H_{48}NO_7P$	$[M-H]^-$
41	LPE 18:1	478.2959	1.696	$C_{23}H_{46}NO_7P$	$[M-H]^-$
42	LPE 18:2	476.2783	1.251	$C_{23}H_{44}NO_7P$	$[M-H]^-$
43	LPE 20:4	500.2808	1.221	$C_{25}H_{44}NO_7P$	$[M-H]^-$
44	LPE 22:5	526.2936	1.505	$C_{27}H_{46}NO_7P$	$[M-H]^-$
45	LPE 22:6	524.2794	1.166	$C_{27}H_{44}NO_7P$	$[M-H]^-$
46	LPE O-16:1	436.2824	1.773	$C_{21}H_{44}NO_6P$	$[M-H]^-$

47	LPE O-16:2	436.2819	1.525	$C_{21}H_{42}NO_6P$	$[M+H]^+$
48	LPE O-18:0	466.3313	2.794	$C_{23}H_{50}NO_6P$	$[M-H]^-$
49	LPE O-18:1	464.3135	2.719	$C_{23}H_{48}NO_6P$	$[M-H]^-$
50	LPE O-18:2	464.3138	2.374	$C_{23}H_{46}NO_6P$	$[M+H]^+$
51	LPI 18:0	599.3221	1.558	$C_{27}H_{53}O_{12}P$	$[M-H]^-$
52	PC 16:0/14:0	706.5394	5.053	$C_{38}H_{76}NO_8P$	$[M+H]^+$
53	PC 16:0/16:0	734.5698	5.703	$C_{40}H_{80}NO_8P$	$[M+H]^+$
54	PC 16:0/16:1	732.5546	5.14	$C_{40}H_{78}NO_8P$	$[M+H]^+$
55	PC 14:0/18:2	730.5365	4.663	$C_{40}H_{76}NO_8P$	$[M+H]^+$
56	PC 15:0/18:1	746.5671	5.45	$C_{41}H_{80}NO_8P$	$[M+H]^+$
57	PC 15:0/18:2	744.5559	4.964	$C_{41}H_{78}NO_8P$	$[M+H]^+$
58	PC 16:0/18:1	760.5846	5.774	$C_{42}H_{82}NO_8P$	$[M+H]^+$
59	PC 16:0/18:2	758.5681	5.277	$C_{42}H_{80}NO_8P$	$[M+H]^+$
60	PC 16:0/18:3	756.5532	4.948	$C_{42}H_{78}NO_8P$	$[M+H]^+$
61	PC 16:1/18:2	756.5529	4.743	$C_{42}H_{78}NO_8P$	$[M+H]^+$
62	PC 18:2/16:3	752.5203	4.414	$C_{42}H_{74}NO_8P$	$[M+H]^+$
63	PC 17:0/18:1	774.602	6.112	$C_{43}H_{84}NO_8P$	$[M+H]^+$
64	PC 17:0/18:2	772.5865	5.597	$C_{43}H_{82}NO_8P$	$[M+H]^+$
65	PC 17:1/18:2	770.5681	5.053	$C_{43}H_{80}NO_8P$	$[M+H]^+$
66	PC 18:0/18:1	788.6179	6.448	$C_{44}H_{86}NO_8P$	$[M+H]^+$
67	PC 18:0/18:2	786.6011	5.931	$C_{44}H_{84}NO_8P$	$[M+H]^+$
68	PC 16:0/20:3	784.586	5.444	$C_{44}H_{82}NO_8P$	$[M+H]^+$
69	PC 18:1/18:2	784.586	5.341	$C_{44}H_{82}NO_8P$	$[M+H]^+$
70	PC 16:0/20:4	782.5664	5.176	$C_{44}H_{80}NO_8P$	$[M+H]^+$
71	PC 18:2/18:2	782.5666	4.869	$C_{44}H_{80}NO_8P$	$[M+H]^+$
72	PC 16:1/20:4	780.5554	4.655	$C_{44}H_{78}NO_8P$	$[M+H]^+$
73	PC 19:0/18:1	802.6337	6.786	$C_{45}H_{88}NO_8P$	$[M+H]^+$
74	PC 19:0/18:2	800.6171	6.268	$C_{45}H_{86}NO_8P$	$[M+H]^+$
75	PC 17:0/20:4	796.5838	5.495	$C_{45}H_{82}NO_8P$	$[M+H]^+$
76	PC 20:0/18:1	816.65	7.127	$C_{46}H_{90}NO_8P$	$[M+H]^+$
77	PC 18:0/20:3	812.6183	6.108	$C_{46}H_{86}NO_8P$	$[M+H]^+$
78	PC 16:0/22:4	810.6018	5.64	$C_{46}H_{84}NO_8P$	$[M+H]^+$
79	PC 18:0/20:4	810.6017	5.817	$C_{46}H_{84}NO_8P$	$[M+H]^+$
80	PC 16:0/22:5	808.5858	5.428	$C_{46}H_{82}NO_8P$	$[M+H]^+$
81	PC 18:1/20:4	808.5862	5.239	$C_{46}H_{82}NO_8P$	$[M+H]^+$
82	PC 16:0/22:6	806.5718	5.02	$C_{46}H_{80}NO_8P$	$[M+H]^+$
83	PC 16:1/22:6	804.5546	4.517	$C_{46}H_{78}NO_8P$	$[M+H]^+$
84	PC 18:0/22:4	838.6305	6.294	$C_{48}H_{88}NO_8P$	$[M+H]^+$
85	PC 18:0/22:5	836.6191	6.083	$C_{48}H_{86}NO_8P$	$[M+H]^+$
86	PC 18:0/22:6	834.6032	5.643	$C_{48}H_{84}NO_8P$	$[M+H]^+$
87	PC 18:1/22:5	834.6036	5.49	$C_{48}H_{84}NO_8P$	$[M+H]^+$
88	PC 18:1/22:6	832.5877	5.069	$C_{48}H_{82}NO_8P$	$[M+H]^+$
89	PC O-16:0/16:0	764.5853	6.137	$C_{40}H_{82}NO_7P$	$[M+HCOO]^-$
90	PE 16:0/16:1	690.5065	5.274	$C_{37}H_{72}NO_8P$	$[M+H]^+$
91	PE 16:0/18:1	718.5402	5.921	$C_{39}H_{76}NO_8P$	$[M+H]^+$
92	PE 16:0/18:2	716.5234	5.412	$C_{39}H_{74}NO_8P$	$[M+H]^+$
93	PE 16:0/18:3	714.5094	5.069	$C_{39}H_{72}NO_8P$	$[M+H]^+$
94	PE 16:1/18:2	712.4933	4.872	$C_{39}H_{72}NO_8P$	$[M-H]^-$
95	PE 17:0/18:1	730.5385	6.269	$C_{40}H_{78}NO_8P$	$[M-H]^-$

96	PE 17:0/18:2	730.5364	5.749	$C_{40}H_{76}NO_8P$	$[M+H]^+$
97	PE 18:0/18:1	746.572	6.592	$C_{41}H_{80}NO_8P$	$[M+H]^+$
98	PE 18:0/18:2	744.5566	6.072	$C_{41}H_{78}NO_8P$	$[M+H]^+$
99	PE 18:1/18:1	742.5385	5.395	$C_{41}H_{78}NO_8P$	$[M-H]^-$
100	PE 16:0/20:3	740.5229	5.596	$C_{41}H_{76}NO_8P$	$[M-H]^-$
101	PE 18:0/18:3	740.5226	5.739	$C_{41}H_{76}NO_8P$	$[M-H]^-$
102	PE 18:1/18:2	742.5371	5.476	$C_{41}H_{76}NO_8P$	$[M+H]^+$
103	PE 16:0/20:4	740.5217	5.306	$C_{41}H_{74}NO_8P$	$[M+H]^+$
104	PE 18:2/18:2	738.5086	5.002	$C_{41}H_{74}NO_8P$	$[M-H]^-$
105	PE 18:0/20:3	770.5678	6.246	$C_{43}H_{80}NO_8P$	$[M+H]^+$
106	PE 16:0/22:4	768.5512	5.776	$C_{43}H_{78}NO_8P$	$[M+H]^+$
107	PE 18:0/20:4	768.5569	5.963	$C_{43}H_{78}NO_8P$	$[M+H]^+$
108	PE 16:0/22:5	766.5362	5.567	$C_{43}H_{76}NO_8P$	$[M+H]^+$
109	PE 18:1/20:4	766.5364	5.374	$C_{43}H_{76}NO_8P$	$[M+H]^+$
110	PE 16:0/22:6	762.5093	5.151	$C_{43}H_{74}NO_8P$	$[M-H]^-$
111	PE 18:0/22:4	796.5837	6.437	$C_{45}H_{82}NO_8P$	$[M+H]^+$
112	PE 18:0/22:5	794.5715	6.225	$C_{45}H_{80}NO_8P$	$[M+H]^+$
113	PE 18:0/22:6	792.5554	5.782	$C_{45}H_{78}NO_8P$	$[M+H]^+$
114	PE 18:1/22:5	790.5393	5.644	$C_{45}H_{78}NO_8P$	$[M-H]^-$
115	PE 18:1/22:6	788.5244	5.218	$C_{45}H_{76}NO_8P$	$[M-H]^-$
116	PE O-16:0/18:1	702.5458	6.372	$C_{39}H_{78}NO_7P$	$[M-H]^-$
117	PE O-18:1/16:1	700.5284	6.264	$C_{39}H_{76}NO_7P$	$[M-H]^-$
118	PE O-16:1/18:2	698.5143	5.734	$C_{39}H_{74}NO_7P$	$[M-H]^-$
119	PE O-18:0/18:1	730.5753	7.057	$C_{41}H_{82}NO_7P$	$[M-H]^-$
120	PE O-18:0/18:2	728.5601	6.526	$C_{41}H_{80}NO_7P$	$[M-H]^-$
121	PE O-18:1/18:1	728.5608	6.946	$C_{41}H_{80}NO_7P$	$[M-H]^-$
122	PE O-18:1/18:2	726.5431	6.409	$C_{41}H_{78}NO_7P$	$[M-H]^-$
123	PE O-16:0/22:4	752.5622	6.208	$C_{43}H_{80}NO_7P$	$[M-H]^-$
124	PE O-10:0/28:5	752.5593	5.971	$C_{43}H_{78}NO_7P$	$[M+H]^+$
125	PE O-16:0/22:5	750.5423	5.99	$C_{43}H_{78}NO_7P$	$[M-H]^-$
126	PE O-18:1/20:4	750.5424	6.288	$C_{43}H_{78}NO_7P$	$[M-H]^-$
127	PE O-16:1/22:5	748.5263	5.875	$C_{43}H_{76}NO_7P$	$[M-H]^-$
128	PE O-16:1/22:6	746.5131	5.429	$C_{43}H_{74}NO_7P$	$[M-H]^-$
129	PE O-18:0/22:4	780.5945	6.888	$C_{45}H_{84}NO_7P$	$[M-H]^-$
130	PE O-18:0/22:5	778.5792	6.7	$C_{45}H_{82}NO_7P$	$[M-H]^-$
131	PE O-18:1/22:6	774.5461	6.091	$C_{45}H_{78}NO_7P$	$[M-H]^-$
132	PG 19:0/17:0	777.5644	6.216	$C_{42}H_{83}O_{10}P$	$[M-H]^-$
133	PI 16:0/18:1	835.5388	4.893	$C_{43}H_{81}O_{13}P$	$[M-H]^-$
134	PI 16:0/18:2	833.5197	4.474	$C_{43}H_{79}O_{13}P$	$[M-H]^-$
135	PI 18:0/18:1	863.5674	5.491	$C_{45}H_{85}O_{13}P$	$[M-H]^-$
136	PI 18:0/18:2	861.5534	5.032	$C_{45}H_{83}O_{13}P$	$[M-H]^-$
137	PI 16:0/20:3	859.5336	4.623	$C_{45}H_{81}O_{13}P$	$[M-H]^-$
138	PI 16:0/20:4	857.5217	4.408	$C_{45}H_{79}O_{13}P$	$[M-H]^-$
139	PI 18:0/20:3	887.5657	5.197	$C_{47}H_{85}O_{13}P$	$[M-H]^-$
140	PI 18:0/20:4	885.553	4.96	$C_{47}H_{83}O_{13}P$	$[M-H]^-$
141	PI 18:0/22:5	911.5714	5.195	$C_{49}H_{85}O_{13}P$	$[M-H]^-$
142	SHexCer 35:2;3O	806.4961	5.324	$C_{41}H_{77}NO_{12S}$	$[M-H]^-$
143	SHexCer 37:2;3O	834.5292	5.981	$C_{43}H_{81}NO_{12S}$	$[M-H]^-$
144	SM 34:0;2O	705.5927	5.198	$C_{39}H_{81}N_2O_6P$	$[M+H]^+$

145	SM 18:1;2O/16:0	703.5749	4.964	$C_{39}H_{79}N_2O_6P$	$[M+H]^+$
146	SM 18:1;2O/18:0	731.6082	5.638	$C_{41}H_{83}N_2O_6P$	$[M+H]^+$
147	SM 18:1;2O/18:1	729.5884	5.066	$C_{41}H_{81}N_2O_6P$	$[M+H]^+$
148	SM 36:3;2O	727.5747	4.595	$C_{41}H_{79}N_2O_6P$	$[M+H]^+$
149	SM 18:1;2O/22:0	787.6684	7.027	$C_{45}H_{91}N_2O_6P$	$[M+H]^+$
150	SM 18:1;2O/24:1	813.6831	6.997	$C_{47}H_{93}N_2O_6P$	$[M+H]^+$
151	ST 24:1;O3;T	498.289	0.47	$C_{26}H_{45}NO_{6S}$	$[M-H]^-$
152	ST 27:1;O;S	465.3039	3.093	$C_{27}H_{46}O_{4S}$	$[M-H]^-$
153	TG 14:0_16:0_16:1	794.7224	10.239	$C_{49}H_{92}O_6$	$[M+NH4]^+$
154	TG 16:0_14:1_16:1	792.7048	9.779	$C_{49}H_{90}O_6$	$[M+NH4]^+$
155	TG 14:0_16:0_18:0	824.7687	11.02	$C_{51}H_{98}O_6$	$[M+NH4]^+$
156	TG 14:0_16:0_18:1	822.7533	10.692	$C_{51}H_{96}O_6$	$[M+NH4]^+$
157	TG 16:0_14:1_18:1	820.7364	10.263	$C_{51}H_{94}O_6$	$[M+NH4]^+$
158	TG 16:0_14:1_18:2	818.7268	9.846	$C_{51}H_{92}O_6$	$[M+NH4]^+$
159	TG 15:0_16:0_18:1	841.7272	10.901	$C_{52}H_{98}O_6$	$[M+Na]^+$
160	TG 15:0_16:1_18:1	839.7066	10.487	$C_{52}H_{96}O_6$	$[M+Na]^+$
161	TG 16:0_16:0_18:0	852.8001	11.408	$C_{53}H_{102}O_6$	$[M+NH4]^+$
162	TG 16:0_16:0_18:1	850.7844	11.022	$C_{53}H_{100}O_6$	$[M+NH4]^+$
163	TG 16:0_16:1_18:1	848.772	10.705	$C_{53}H_{98}O_6$	$[M+NH4]^+$
164	TG 16:0_16:0_18:3	851.7046	10.39	$C_{53}H_{96}O_6$	$[M+Na]^+$
165	TG 16:0_16:1_18:2	846.7498	10.238	$C_{53}H_{96}O_6$	$[M+NH4]^+$
166	TG 16:0_16:1_18:3	849.6956	10.023	$C_{53}H_{94}O_6$	$[M+Na]^+$
167	TG 16:1_16:1_18:2	844.738	9.869	$C_{53}H_{94}O_6$	$[M+NH4]^+$
168	TG 16:0_17:0_18:1	864.8044	11.309	$C_{54}H_{102}O_6$	$[M+NH4]^+$
169	TG 16:0_17:1_18:1	862.7853	10.832	$C_{54}H_{100}O_6$	$[M+NH4]^+$
170	TG 15:0_18:1_18:2	860.7682	10.464	$C_{54}H_{98}O_6$	$[M+NH4]^+$
171	TG 16:0_18:0_18:1	878.8129	11.411	$C_{55}H_{104}O_6$	$[M+NH4]^+$
172	TG 16:0_18:1_18:1	876.7953	11.031	$C_{55}H_{102}O_6$	$[M+NH4]^+$
173	TG 16:0_18:1_18:2	874.7846	10.678	$C_{55}H_{100}O_6$	$[M+NH4]^+$
174	TG 16:0_18:1_18:3	872.7708	10.486	$C_{55}H_{98}O_6$	$[M+NH4]^+$
175	TG 16:1_18:1_18:2	872.7682	10.291	$C_{55}H_{98}O_6$	$[M+NH4]^+$
176	TG 16:0_18:2_18:3	870.7525	10.005	$C_{55}H_{96}O_6$	$[M+NH4]^+$
177	TG 16:1_18:1_18:3	870.7512	9.892	$C_{55}H_{96}O_6$	$[M+NH4]^+$
178	TG 16:1_18:2_18:3	873.6916	9.56	$C_{55}H_{94}O_6$	$[M+Na]^+$
179	TG 17:0_17:0_19:1	892.8315	11.503	$C_{56}H_{106}O_6$	$[M+NH4]^+$
180	TG 17:0_18:1_18:1	890.8141	11.23	$C_{56}H_{104}O_6$	$[M+NH4]^+$
181	TG 17:0_18:1_18:2	888.7979	10.877	$C_{56}H_{102}O_6$	$[M+NH4]^+$
182	TG 17:1_18:1_18:2	886.7839	10.574	$C_{56}H_{100}O_6$	$[M+NH4]^+$
183	TG 18:0_18:0_18:1	906.8482	11.769	$C_{57}H_{108}O_6$	$[M+NH4]^+$
184	TG 18:0_18:1_18:1	904.8279	11.418	$C_{57}H_{106}O_6$	$[M+NH4]^+$
185	TG 18:0_18:1_18:2	907.7728	11.156	$C_{57}H_{104}O_6$	$[M+Na]^+$
186	TG 18:1_18:1_18:1	902.8151	11.05	$C_{57}H_{104}O_6$	$[M+NH4]^+$
187	TG 18:1_18:1_18:2	900.8016	10.693	$C_{57}H_{102}O_6$	$[M+NH4]^+$
188	TG 16:0_18:1_20:4	898.7803	10.555	$C_{57}H_{100}O_6$	$[M+NH4]^+$
189	TG 18:1_18:2_18:2	898.7889	10.406	$C_{57}H_{100}O_6$	$[M+NH4]^+$
190	TG 16:0_18:2_20:4	896.7664	10.25	$C_{57}H_{98}O_6$	$[M+NH4]^+$
191	TG 18:1_18:2_18:3	896.7656	9.971	$C_{57}H_{98}O_6$	$[M+NH4]^+$
192	TG 18:1_18:1_20:1	930.85	11.497	$C_{59}H_{108}O_6$	$[M+NH4]^+$
193	TG 16:0_18:0_22:4	933.7924	11.322	$C_{59}H_{106}O_6$	$[M+Na]^+$

194	TG 18:0_18:1_20:3	933.782	11.176	$C_{59}H_{106}O_6$	$[M+Na]^+$
195	TG 16:0_18:1_22:4	926.8231	10.856	$C_{59}H_{104}O_6$	$[M+NH4]^+$
196	TG 18:0_18:1_20:4	926.8137	10.983	$C_{59}H_{104}O_6$	$[M+NH4]^+$
197	TG 18:1_18:2_20:2	931.7709	10.782	$C_{59}H_{104}O_6$	$[M+Na]^+$
198	TG 16:0_18:1_22:5	924.8022	10.549	$C_{59}H_{102}O_6$	$[M+NH4]^+$
199	TG 16:0_18:1_22:6	922.7831	10.389	$C_{59}H_{100}O_6$	$[M+NH4]^+$
200	TG 18:1_18:2_20:4	922.7849	10.264	$C_{59}H_{100}O_6$	$[M+NH4]^+$
201	TG O-18:0_18:1_18:1	890.8362	11.229	$C_{57}H_{108}O_5$	$[M+NH4]^+$
